# No Correlation between Distorted Body Representations Underlying Tactile Distance Perception and Position Sense

**DOI:** 10.3389/fnhum.2016.00593

**Published:** 2016-11-21

**Authors:** Matthew R. Longo, Rosa Morcom

**Affiliations:** Department of Psychological Sciences, Birkbeck, University of LondonLondon, UK

**Keywords:** position sense, tactile distance, body represenation, body image, body schema

## Abstract

Both tactile distance perception and position sense are believed to require that immediate afferent signals be referenced to a stored representation of body size and shape (the *body model*). For both of these abilities, recent studies have reported that the stored body representations involved are highly distorted, at least in the case of the hand, with the hand dorsum represented as wider and squatter than it actually is. Here, we investigated whether individual differences in the magnitude of these distortions are shared between tactile distance perception and position sense, as would be predicted by the hypothesis that a single distorted body model underlies both tasks. We used established tasks to measure distortions of the represented shape of the hand dorsum. Consistent with previous results, in both cases there were clear biases to overestimate distances oriented along the medio-lateral axis of the hand compared to the proximo-distal axis. Moreover, within each task there were clear split-half correlations, demonstrating that both tasks show consistent individual differences. Critically, however, there was no correlation between the magnitudes of distortion in the two tasks. This casts doubt on the proposal that a common body model underlies both tactile distance perception and position sense.

## Introduction

Several forms of perception require that immediate sensory signals be combined with stored representations of body size and shape. This need is most acute in somatosensation, for which the primary receptor surface—the skin—is physically co-extensive with the body itself. We recently proposed a model of somatoperceptual information processing which postulated a common representation of the metric properties of the body (the *body model*) underlying perceptual abilities such as tactile distance perception and position sense (Longo et al., 2010). In the case of touch, several recent studies have shown that illusions and similar interventions which alter the perceived size of body parts produce corresponding changes in the perceived size of objects touching those parts, including effects induced by visual magnification (Taylor-Clarke et al., 2004), proprioceptive illusions (de Vignemont et al., 2005), cutaneous anesthesia (Berryman et al., 2006), the rubber hand illusion (Haggard and Jundi, 2009; Bruno and Bertamini, 2010), action sounds (Tajadura-Jiménez et al., 2012, 2015), and tool use (Canzoneri et al., 2013; Miller et al., 2014). Together, such results support the interpretation that the perception of tactile distance involves immediate tactile signals being referenced to higher-order models of the size and shape of the body.

Other studies have investigated the body representations underlying both tactile distance perception and position sense at baseline, in the absence of any manipulation of perceived bodily form. For example, Weber (1834/1996) in his classic studies on touch found that as he moved the two points of a compass across his skin, it felt like the distance between the two points increased as he moved them from a region or relatively low spatial sensitivity (e.g., the upper arm) to a region of higher sensitivity (e.g., the palm). Subsequent studies have replicated this general pattern, showing that perceived tactile distances appear to be systematically related to the sensitivity of different skin surfaces (e.g., Goudge, 1918; Marks et al., 1982; Cholewiak, 1999; Taylor-Clarke et al., 2004; Anema et al., 2008; Miller et al., 2016). Similarly, large anisotropies of perceived tactile distance have been reported on the limbs, with stimuli oriented across the width of limbs being perceived as substantially farther apart than stimuli oriented along the length of the limbs (Green, 1982; Longo and Haggard, 2011; Canzoneri et al., 2013; Longo and Sadibolova, 2013; Le Cornu Knight et al., 2014; Miller et al., 2014, 2016; Longo, 2015). For example, in the study of Longo and Haggard (2011), we presented participants sequentially with two pairs of touches on each trial, one pair oriented along the proximo-distal axis of their hand and the other oriented with the medio-lateral axis. Across trials, the ratio of the distances in the medio-lateral and proximo-distal orientations was manipulated according to the method of constant stimuli. Participants were asked to make two-alternative forced-choice (2AFC) judgments of which of the two stimuli had a larger distance between the two touches. We then estimated the point-of-subjective-equality (PSE) for each participant, finding a clear bias to overestimate the distance between touches oriented with the medio-lateral hand axis.

In the case of position sense, recent studies have provided evidence for similar distortions. Longo and Haggard (2010) developed a method to isolate and measure the stored body representation which is integrated with immediate afferent signals. In this task, participants sit with their hand underneath an occluding board and are asked to judge the perceived location of the tips and knuckles of each finger. By comparing the relative locations of judgments of each landmark, an implicit perceptual map of the hand can be constructed and compared to the actual form of the hand. Studies using this paradigm have revealed a fat, squat hand representation, with overestimation of hand width and underestimation of finger length (e.g., Longo and Haggard, 2010, 2012a,b; Lopez et al., 2012; Ferrè et al., 2013; Longo, 2014, 2015; Mattioni and Longo, 2014; Coelho et al., 2016; Saulton et al., 2016).

Thus, large and highly stereotyped distortions have been reported for both tactile distance perception and position sense. What is the relation between body representations underlying these two abilities? In the model of somatoperceptual information processing proposed by Longo et al. (2010), a common body model feeds into both of these perceptual processes. Evidence consistent with the proposal that a common body model underlies both tactile distance perception and position sense comes from findings of similar patterns of distortions for both forms of perception. For example, as discussed above, there are clear biases to overestimate the width of the hand compared to its length, both in tactile distance perception (e.g., Green, 1982; Longo and Haggard, 2011) and in position sense (e.g., Longo and Haggard, 2010). Further, distortions are substantially larger on the hairy skin of the hand dorsum than on the glabrous skin of the palm, both for tactile distance perception (Longo and Haggard, 2011; Le Cornu Knight et al., 2014; Longo et al., 2015a,b) and position sense (Longo and Haggard, 2012a).

The present study investigated whether a common body model underlies tactile distance perception and position sense by looking at whether individual differences in the magnitude of distortions are shared between these abilities. In previous research, strong correlations have been found between the magnitude of distortion on the two hands and across similar conditions for both tactile size perception (Longo et al., 2015a) and position sense (Longo and Haggard, 2010, 2012a; Longo, 2014; Mattioni and Longo, 2014). Thus, it is clear that reliable individual differences exist for both perceptual abilities. Here we investigated whether these individual differences are shared across abilities by measuring both in the same people. We measured anisotropy of tactile distance perception on the dorsum of the left hand using a two-alternative forced-choice (2AFC) method similar to that we have used previously (Longo and Haggard, 2011; Longo et al., 2015a). Because the method described above for producing proprioceptive maps underlying position sense focuses on the fingers, we used a revised procedure we recently reported (Longo et al., 2015b) which allows mapping the hand dorsum. Specifically, instead of giving participants verbal instructions about which landmark to localize, a point on the hand is touched and participants are asked to localize the touch in external space. This allows proprioceptive maps to be constructed even for regions of skin without lexically-labeled landmarks. Indeed, we found that these maps were stretched along the medio-lateral hand axis (Longo et al., 2015b). If a common body model underlies both tactile distance perception and position sense, we expected a correlation across participants in the magnitude of the distortions found for each task.

## Materials and Methods

### Participants

Twenty-five members of the Birkbeck community (18 females; mean age: 30.5 years, SD: 8.8 years) participated after giving informed consent. All participants were right-handed as assessed by the Edinburgh Inventory (Oldfield, 1971; *M*: 82.5, range: 36.8–100). Five additional participants with an *R*^2^ lower than 0.50 on at least one of the two blocks of the tactile distance task were excluded from analyses. The relatively high exclusion rate is largely driven by the fact that participants needed to have good fit to their data in both of the halves of the experiment. Had the same criteria been applied to the complete set of data from each participant, only two participants would have been excluded.

All procedures were approved by the Department of Psychological Sciences Research Ethics Committee at Birkbeck, University of London. The study was conducted in accordance with the principles of the Declaration of Helsinki.

### Tactile Distance Task

Procedures for the tactile distance task were similar to our previous studies using this paradigm (Longo and Haggard, 2011; Longo and Sadibolova, 2013; Longo et al., 2015a). Stimuli were pairs of wooden posts mounted in foamboard, separated by 2, 3, or 4 cm. The posts tapered to a blunt point (approximately 1 mm in diameter). On each trial, participants were touched twice on the dorsal surface of their left hand, once with the posts oriented across the medio-lateral hand axis (*across* orientation), and once with the posts oriented along the proximo-distal hand axis (*along* orientation). Participants made untimed 2AFC judgments of which of the two distances felt physically larger. Stimuli were applied manually by an experimenter, approximately in the center of the hand dorsum. Stimuli lasted approximately 1 s with an approximately 1 s inter-stimulus interval.

There were two blocks of 72 trials each. In each block, all nine combinations of across and along stimuli were presented eight times each, in random sequence. The order of the along and across stimuli was counterbalanced across trials. The two blocks were separated by a short break. Participants were blindfolded throughout the procedure.

The percentage of trials in which the “across” stimulus was judged as larger was analyzed as a function of the ratio of the length of the across and along stimuli, plotted logarithmically to produce a symmetrical distribution around a ratio of 1 (i.e., the point-of-actual-equality). Best-fitting cumulative Gaussian functions were fit to data from individual participants using maximum-likelihood estimation with the Palamedes toolbox (Prins and Kingdom, 2009) for MATLAB (Mathworks, Natick, MA, USA).

For each participant, the point-of-subjective-equality (PSE) was quantified as the mean of the best-fitting Gaussian. As mentioned above, data for five participants was excluded because the *R*^2^ of the best-fitting Gaussian fit separately to Blocks 1 and 2 was below 0.50 in at least one block. For the remaining participants, there was good fit for the data with mean *R*^2^ of 0.930 overall, 0.878 for curves fit to Block 1, and 0.873 for curves fit to Block 2.

### Proprioceptive Maps

Procedures were similar to those in our recent article (Longo et al., 2015b). Participants sat with their left hand resting palm down on a table. The hand rested flat on the table, with fingers completely straight. An occluding board (40 cm × 40 cm) was placed over the hand, resting on four pillars (6 cm high). A camera (Logitech Webcam Pro 9000 HD) suspended on a tripod above the occluding board (27 cm high) captured photographs (1600 × 1200 pixels) controlled by a custom MATLAB script.

**Figure 1:**
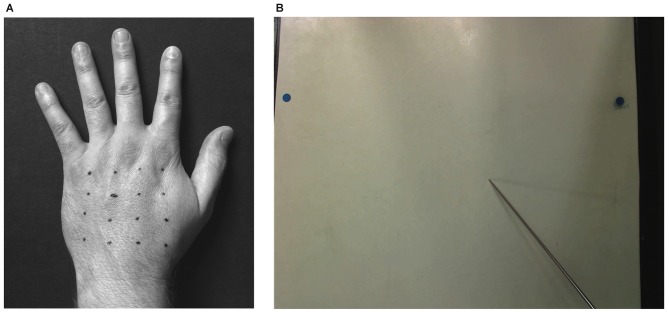
**Setup of the proprioceptive mapping task. (A)** A 4 × 4 grid of locations was marked with pen on the back of the participant’s hand. **(B)** On each trial, one of these locations was touched and participants used a long baton to judge the perceived location at which the touch had occurred by pointing to the corresponding location on an occluding board covering their hand. Locations of responses were captured by an overhead camera.

To identify the points of stimulation, a 4 × 4 grid of points was marked with a pen on the back of the participant’s hand using a plastic template (see Figure 1A). The four rows of points ran along the medio-lateral hand axis, while the four columns ran along the proximo-distal axis. On each trial, the experimenter lifted the occluding board (turning it towards the participant so that it still blocked their view of their hand), and touched one of the points with a von Frey hair (255 milliNewtons) for approximately 1 s. The participant’s task was to place the tip of a long baton (35 cm length, 2 mm diameter) on the occluder directly above the location where the touch had occurred. They were instructed to be precise in their judgments and avoid ballistic pointing or strategies such as tracing the outline of the hand. To ensure that they judged each landmark individually, participants moved the baton to the edge of the board before the start of each trial. When the participants indicated their response, a photograph was taken and saved for offline coding (see Figure 1B).

There were four blocks of 48 trials each. Each block included three mini-blocks of one repetition of each of the 16 stimulus locations in random order. At the beginning and the end of each block a photograph of the participant’s hand was taken to measure the true locations of the applied stimuli and to check that the hand hadn’t moved during the course of the block. A 10 cm ruler appeared in the photographs of the participant’s hand and allowed conversion between pixel units and centimeters.

For offline data coding, the *x-y* pixel coordinates of each landmark were coded using a custom MATLAB script using Cogent Graphics (developed by John Romaya, Wellcome Department of Imaging Neuroscience, University College London). Mean coordinates were then calculated for each location in each experimental block. The set of mean coordinates in each block comprises two maps, one reflecting the actual shape of the stimulated locations, the other reflecting represented shape. Distances between mean pixel coordinates of pairs of locations differing in the medio-lateral and proximo-distal orientations were calculated and converted into cm. As shown in Figure 3, three types of distances were calculated in each orientation: *small* distances, between adjacent locations; *mid* distances, between locations separated by a single other location; and *large* distances, separated by two other locations. There were 12 small, 8 mid, and 4 large distances in each orientation.

To assess overall stretch of maps, we stretched an idealized square grid reflecting the locations of the 16 points by different amounts to find the stretch that maximized the similarity with each participant’s perceptual map, as well as with the actual configuration of points on their hand. Stretches were defined by the multiplication of the *x*-coordinate (reflecting location in the medio-lateral hand axis) by a stretch parameter. Thus, a stretch of 1 indicated a perfectly square grid, stretch of less than 1 indicated a tall thin grid, and stretch of more than 1 indicated a squat fat grid. Values between 0.33 and 3 were tested by exhaustive search with a resolution of 0.0005 units in natural logarithm space (i.e., 4415 steps).

## Results

### Tactile Distance Task

**Figure 2:**
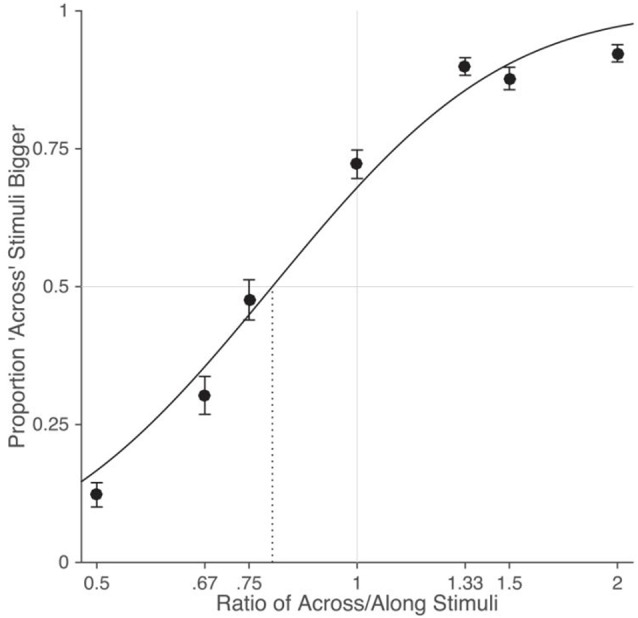
**Results from the tactile distance judgment task.** As in previous studies, there was a clear bias for stimuli oriented across the width of the hand dorsum to be perceived as larger than stimuli oriented along the length of the hand. The dotted vertical line indicates the point-of-subjective equality (i.e., the stimulus ratio at which participants were equally likely to judge the across or the along stimulus as bigger). Error bars are one standard error.

Results from the tactile distance judgment task are shown in Figure 2. Psychometric functions were fit to the data from each participant and the PSE was calculated as the ratio between the across and along stimuli where the curve crossed 50% (i.e., the ratio for which the participant was equally likely to judge the across or the along stimulus as bigger). There was a clear bias to perceive distances across the width of the hand as bigger than those along the length of the hand (*M*: 0.782), *t*_(24)_ = −9.79, *p* < 0.0001, *d* = 1.96. This clearly replicates the anisotropy reported previously (Green, 1982; Longo and Haggard, 2011; Canzoneri et al., 2013; Longo and Sadibolova, 2013; Le Cornu Knight et al., 2014; Miller et al., 2014; Longo et al., 2015a).

### Proprioceptive Maps

**Figure 3:**
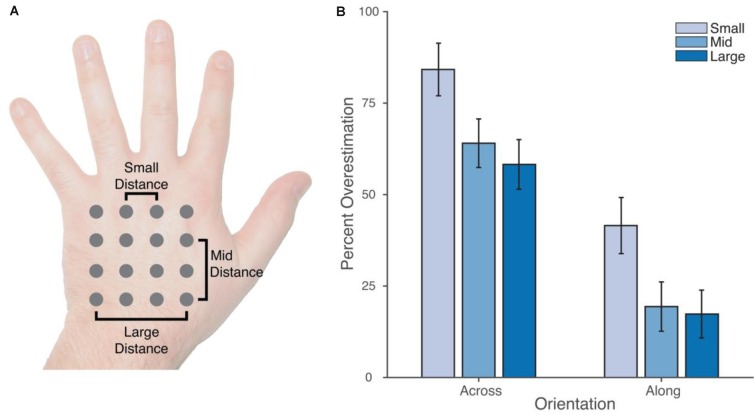
**(A)** Schematic depiction of the location of the 16 stimulus locations in the proprioceptive map task. Three types of distance were calculated in both the across and along orientations, reflecting one step between locations (*small distance*), two steps (*mid distance*), or three steps (*large distance*). **(B)** Overestimation of distances in the two orientations as a percentage of actual distance. While absolute overestimation was apparent in both orientations, it was substantially larger in the across than in the along orientation. Error bars are one standard error.

To quantify distortions in the internal configuration of the representation of the hand, we calculated the distance between judgments of pairs of locations differing in location along the medio-lateral hand axis (across the hand) or the proximo-distal axis (along the hand), as shown in the Figure 3A. Distances across the hand were calculated for pairs of landmarks within each row of locations, and distances along the hand were calculated for pairs of landmarks within each column. Three sizes of distance were calculated: *small* distances, separated by a single step; *mid* distances, separated by two steps; and *large* distances, separated by two steps. There were, thus, 12 small, 8 mid, and 4 large distances in each orientation.

Figure 3B shows overestimation as a percentage of actual distance for across and along distances of the three different sizes. One-sample *t*-tests with Holm-Bonferroni correction for multiple comparisons were used to compare the amount of overestimation to 0. Significant overestimation was found for all distances, both across the hand: *small* (*M* = 84.2%, *t*_(24)_ = 11.71, *p* < 0.0001, *d* = 2.41), *mid* (*M* = 64.0%, *t*_(24)_ = 9.65, *p* < 0.0001, *d* = 2.01), *large* (*M* = 58.2%, *t*_(24)_ = 8.59, *p* < 0.0001, *d* = 1.78); and along the hand: *small* (*M* = 41.5%), *t*_(24)_ = 5.41, *p* < 0.0001, *d* = 1.12; *mid* (*M* = 19.4%), *t*_(24)_ = 2.88, *p* < 0.02, *d* = 0.60; *large* (*M* = 17.3%), *t*_(24)_ = 2.65, *p* < 0.02, *d* = 0.55. Critically, however, the magnitude of overestimation was significantly larger in the across than in the along orientation in all cases: *small*, *t*_(24)_ = 6.26, *p* < 0.0001, *d_z_* = 1.31; *mid*, *t*_(24)_ = 7.40, *p* < 0.0001, *d_z_* = 1.54; *large*, *t*_(24)_ = 7.73, *p* < 0.0001, *d_z_* = 1.61.

A 2 × 3 repeated-measures analysis of variance (ANOVA) was conducted with orientation (across vs. along) and size (small, medium, large) as factors. There was a clear main effect of orientation, *F*_(1,24)_ = 63.71, *p* < 0.0001, $\eta_{p}^{2}$ = 0.73, with distances across the width of the hand overestimated relative to those along the length of the hand. There was also a main effect of size, *F*_(1.21,29.09)_ = 34.77, *p* < 0.0001, $\eta_{p}^{2}$ = 0.59, with overestimation decreasing monotonically with size. There was no interaction, *F*_(1.62,38.80)_ = 0.439, *n.s*., $\eta_{p}^{2}$ = 0.02.

**Figure 4:**
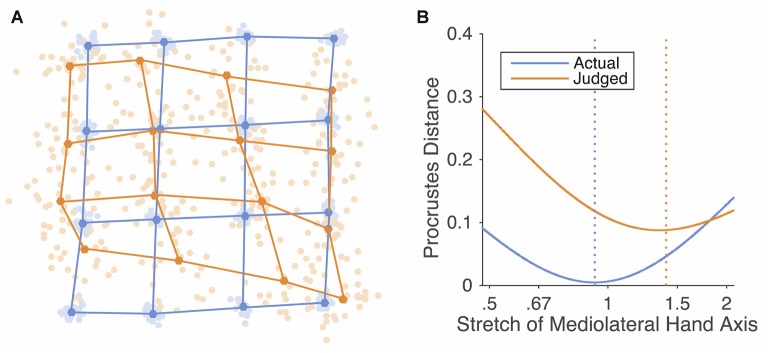
**(A)** Generalized Procrustes alignment of the actual configuration of points on the hand (blue dots and lines) and perceptual maps (orange dots and lines). The light dots are data from individual participants, while the dark dots represent the average shape. **(B)** Mean Procrustes distance between actual and perceptual maps and idealized grids stretched by different amounts. A stretch of 1 indicates a square grid; stretches greater than 1 indicate stretch in the medio-lateral axis, while stretches less than 1 indicate stretch in the proximo-distal axis.

The analyses reported so far calculate separate measures of overestimation for each dimension. To calculate a single measure of distortion of maps as a whole, we conducted an additional analysis using a method called *Procrustes alignment* (Rholf and Slice, 1990; Bookstein, 1991). Procrustes alignment superimposes configurations of homologous landmarks by translating, scaling, and rotating them so as to minimize the distance between pairs of landmarks. We used this in two ways. First, we used Generalized Procrustes Analysis (Gower, 1975) to mutually superimpose maps from all participants to construct grand-averages of both perceptual maps and actual hand shape. These maps are shown in the Figure 4A and allow a visualization of the overall pattern of distortions.

Second, we used the Procrustes distance, the sum-of-squares of the residual distances between pairs of homologous landmarks, as a measure of the dissimilarity between two maps. This allowed us to estimate the overall stretch of perceptual maps in the medio-lateral axis by finding the stretch applied to an idealized rectangular grid that minimized the dissimilarity with each map. We multiplied the *x*-coordinates of a 4 × 4 rectangular grid by a stretch parameter to generate grids of varying levels of stretch. When the stretch parameter was equal to 1, the grid was perfectly square. When it was greater than 1, the grid was stretched in the medio-lateral axis. When it was less than 1, the grid was stretched in the proximo-distal axis. For each participant, we determined the value of the stretch parameter that minimized the dissimilarity in shape (i.e., that minimized the Procrustes distance) between the stretched grid and the participant’s perceptual map. Figure 4B shows the mean values of the Procrustes distance for values of the stretch parameter. The best-fitting stretch parameters were significantly greater than 1 (*M*: 1.40), *t*_(24)_ = 6.96, *p* < 0.0001, *d* = 1.39.

### Correlations Between Tasks

**Figure 5:**
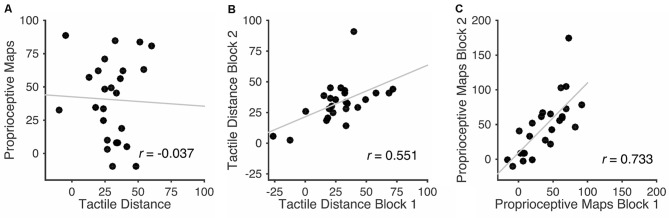
**Scatterplots showing the relation between the two tasks (A)**, and between the first and second halves of each of the two tasks individually **(B,C)**. Units are percent overestimation of the medio-lateral relative to the proximo-distal hand axis. There was no correlation between the magnitude of the distortion in the two tasks. Critically, however, within each task, clear split-half correlations were apparent.

Figure 5A shows a scatterplot of distortions in the two tasks, in both cases quantified as the percentage overestimation of the medio-lateral hand axis relative to the proximo-distal axis. There was no apparent relationship whatsoever, with a highly non-significant correlation, *r*_(23)_ = −0.037, *p* = 0.861.

To ensure that this lack of correlation does not reflect an absence of meaningful individual differences in these measures or a lack of statistical power, we investigated the split-half correlations between the two blocks of each task. A scatterplot showing the relation between performance on the two blocks of the tactile distance judgment task is shown in the Figure 5B, and a corresponding scatterplot for the proprioceptive localization task in the Figure 5C. As is clear in the Figure, clear split-half correlations were apparent in both the tactile distance, *r*_(23)_ = 0.551, *p* < 0.005, and proprioceptive localization, *r*_(23)_ = 0.733, *p* < 0.0001, tasks.

## Discussion

These results replicated the distortions that have previously been reported on the hand dorsum for both tactile distance perception (see Green, 1982; Longo and Haggard, 2011) and position sense (Longo and Haggard, 2010, 2012a). Moreover, also consistent with previous results, there were clear individual differences in the magnitude of these distortions, as measured by split-half correlations. Critically, however, there was no evidence that individual differences were shared between tactile distance perception and position sense, with no apparent correlation between distortions in the two cases. These results cast doubt on the suggestion that both abilities rely on a common representation of the body’s metric properties (i.e., body model), as we suggested previously (Longo et al., 2010), in which case common individual differences should be apparent in both cases.

What causes individual differences in these tasks? To this point, we have assumed that the split-half correlations we find reflect differences between people in the extent to which the representation of the hand’s metric properties (i.e., the body model) is distorted. It is certainly possible, however, that these correlations might instead reflect differences between people in the way they approach the task or the amount of effort they exert. Given that the tasks we used are superficially very different, person-to-person differences in how the tasks are approached might affect the tasks in different ways. Thus, it is possible that the overall similar distortions in the two tasks seen at the level of the overall mean reflect the influence of a common body model on both tasks, but that the split-half correlations reflect idiosyncratic differences in how participants approach each task. The present results cannot exclude this interpretation. However, in a recent study (Longo et al., 2015a) we found that while there were clear correlations in the magnitude of distortions of tactile distance perception across the two hands, there were no correlations between distortions on the palm and dorsum of each hand. Given that the task was exactly the same for both skin surfaces, the lack of correlation between the palm and dorsum is difficult to interpret in terms of how participants approached the task.

What do the present results tell us about the relation between distortions in tactile distance perception and position sense? In both cases, the nature of the distortions appears to parallel lower-level aspects of somatosensory organization. For example, the overestimation of hand width relative to length mirrors findings of greater tactile spatial acuity in the medio-lateral than in the proximo-distal axis of the limbs (e.g., Weber, 1834/1996; Cody et al., 2008) and the fact that receptive fields of neurons in the spinal cord and cortex representing the limbs tend to be oval-shaped, with the long axis running along the proximo-distal limb axis (e.g., Powell and Mountcastle, 1959; Brooks et al., 1961; Brown et al., 1975; Alloway et al., 1989). Distortions in both tactile distance perception and position sense, however, are much smaller than would be predicted on the basis of receptive field size alone (Taylor-Clarke et al., 2004; Longo, 2017), suggesting that low-level distortions are at least partly corrected before affecting tactile distance perception and position sense. Thus, one possibility is that body representations underlying tactile distance perception and position sense are completely distinct, but both are shaped by lower-level somatosensory maps, and inherit their distortions. This could account for the fact that both perceptual abilities show qualitatively similar patterns of distortion, which are nevertheless not correlated across people. Another possibility is that both tactile distance perception and position sense rely on a common body model, but that the specific demands of each type of judgment alter responses, resulting in different patterns of individual difference in the two cases. The present results do not exclude either of these possibilities.

The procedure for mapping implicit body representations developed by Longo and Haggard (2010) relies on the body part being mapped having numerous distinct landmarks with verbally-specifiable names. This worked in the case of the hands, which have many such lexically-coded landmarks, at least on the fingers. Together with a recent study (Longo et al., 2015b), the present results show that this paradigm can be extended to regions of the body which do not have such landmarks. In both of these studies, perceptual maps analogous to those obtained by Longo and Haggard (2010) were obtained for the hand dorsum, which (unlike the fingers) lacks many distinct landmarks. Critically, these maps showed overestimation of hand width relative to length, analogous to the underestimation of finger length and overestimation of hand width described by Longo and Haggard (2010). This demonstrates that the distortions seen in previous studies cannot be an artifact of the use of verbal categories for cueing responses. That implicit perceptual maps can be obtained in the absence of distinct landmarks also allows the possibility of mapping regions of the body beyond the hands.

## Author Contributions

MRL designed the study, analyzed the data, and wrote the article. RM collected the data and analyzed the data.

## Conflict of Interest Statement

The authors declare that the research was conducted in the absence of any commercial or financial relationships that could be construed as a potential conflict of interest.
